# Comparison between unilateral biportal endoscopic and percutaneous posterior endoscopic cervical keyhole surgery for patients with cervical spondylotic radiculopathy

**DOI:** 10.3389/fsurg.2026.1774968

**Published:** 2026-05-20

**Authors:** Bo Zhu, Hailong Chen

**Affiliations:** Department of Spine, Luoyang Orthopedic-Traumatological Hospital of Henan Province (Henan Provincial Orthopedic Hospital), Luoyang, China

**Keywords:** clinical outcomes, comparative study, percutaneous endoscopic cervical keyhole foraminotomy, unilateral biportal endoscopic, unilateral cervical radiculopathy

## Abstract

**Study design:**

The present study was retrospective in design.

**Objective:**

The aim of this study was to compare the clinical outcomes of unilateral biportal endoscopic (UBE) and percutaneous posterior endoscopic cervical (PE) keyhole foraminotomy surgeries for patients with cervical spondylotic radiculopathy (CSR).

**Background data:**

A total of 123 eligible patients with single-level cervical spine disc herniation treated with UBE or PE at our hospital between April 2020 and April 2023 were included. Patients were divided into two groups according to operation procedure. There were 28 men and 32 women in the UBE group with a mean age of 55.0 ± 9.0 years, while there were 29 men and 34 women in the PE group with a mean age of 57.3 ± 6.9 years.

**Method:**

Data were collected from medical records. The study evaluated baseline characteristics, operation-related parameters (hospital stay, cost, and blood loss), clinical outcomes (Visual Analogue Scale [VAS] and Neck Disability Index [NDI]), and perioperative data (operation time, cost, surgery level, and pain level). MacNab criteria grade and complications were evaluated and compared.

**Result:**

There were no significant differences in age, gender, pain level, operation level, preoperative VAS, NDI, and C2/7 Cobb angle between the two groups (*P* > 0.05). The UBE group showed significantly lower fluoroscopy and operation times. Moreover, patients in the UBE group achieved significantly better improvement in NDI, VAS-N (VAS of neck pain), and VAS-A (VAS of arm pain) at each follow-up visit compared with the PE group (*P* < 0.05). The excellent/good rates (MacNab criteria) in the UBE and PE groups were 96.67% (58) and 92.06% (58), respectively. In the PE group, three patients suffered from nerve root irritation and four sustained mild spinal epidural tears; in contrast, only one patient in the UBE group experienced nerve root irritation.

**Conclusion:**

UBE may provide better clinical outcomes than PE in patients with CSR. More studies need to be conducted to further confirm the efficacy of UBE.

## Introduction

With an aging population, cervical radiculopathy has become an increasingly common condition frequently encountered in clinical practice. A recently published article showed that cervical radiculopathy prevalence ranges from 1.07–1.76 per 1,000 for males and 0.63–5.8 per 1,000 for females (1). The pathology of CSR is intervertebral foramen stenosis caused by cervical degenerative changes which can lead to neurothlipsis, such as lateral disc herniation and osteophytes (2). As the main symptoms of CSR are neck and arm pain, finger numbness, and arm weakness, complete nerve depression and pain relief are the main aims in cervical radiculopathy treatment (3). Surgical treatment is always needed for complete decompression and to avoid degenerative progression which conservative treatment cannot achieve.

Adamson et al. reported on 100 patients who were diagnosed with lateral canal or foraminal compression by radiological exam and underwent percutaneous endoscopic cervical keyhole foraminotomy (PE); 97 patient reported excellent or good results according to the MacNab criteria (4). However, numerous complications were reported in patients who underwent PE. Gou et al. reported that complications occurred in 40 of the 701 patients who underwent percutaneous endoscopic cervical keyhole foraminotomy. The incidence of total complications in the PE group was 4.7% (95% CI, 2.9%–7.0%) (5). Lou et al. reported on 33 patients with CSR underwent keyhole foraminotomy via a percutaneous full endoscopic approach; of these, two patients reported transient postoperative dysesthesia and dural tear during operation (6). Similarly, Ruetten et al. reported on three patients who suffered from intransigent dermatoma-related hypesthesia who underwent keyhole foraminotomy via a percutaneous full endoscopic approach (7).

In 1996, Antoni et al. first reported the unilateral biportal endoscopy (UBE) technique, which is recognized for providing greater instrument flexibility, superior decompression, and fewer intraoperative fluoroscopic procedures (8, 9). Song et al. reported that continuous saline irrigation made UBE safer for posterior cervical inclinatory foraminotomy for cervical radiculopathy (10). Kang et al. reported that the UBE technique can significantly reduce the operation time with a senior surgeon proficient in cervical keyhole surgery (11). However, complications such as spinal cord injury have also been reported in patients who undergo UBE procedure (12).

So far, there have been few studies comparing the clinical outcomes between PECF and UBE and exploring whether UBE can improve clinical recovery and diminish complications. Therefore, our study aimed to analyze the therapeutic efficacy of these two methods and test whether UBE for patients with cervical radiculopathy can (1) provide better pain relief and cervical motion, (2) decrease complications after operation, and (3) allow patients better outcomes during follow-up.

## Method

### Patients

This retrospective analysis was conducted using data from a prospectively collected, single-center database that included patients treated with PE or UBE at our hospital from April 2020 to April 2023. The study population consisted of adult patients older than 25 who underwent surgical treatment for single-level unilateral cervical radiculopathy.

All data were collected from medical records. Cases were diagnosed based on clinical symptoms, physical examinations, and MRI, following the failure of at least three months of conservative treatment. The inclusion criteria were patients (1) aged >25 with single-level unilateral cervical radiculopathy, (2) who had failed palliative therapy for at least 3 months, (3) who were treated with PE or UBE with a minimal 1-year follow-up visit. The exclusion criteria were patients (1) with a previous surgical history involving the cervical spine; (2) with cervical abnormalities including hemivertebrae, block vertebrae, or butterfly vertebrae; and (3) with other comorbidities that may impact clinical outcomes, such as tumor, rheumatic arthritis, tuberculosis, or chronic renal failure.

The present study followed the Declaration of Helsinki. Informed consent was obtained from all participants, who were informed that their information would be stored anonymously and used for research, and this study was approved by the Institutional Review Boards and Ethics Committee of our hospital.

### Surgery procedure

All operations were performed by the same chief spine surgeon. Details on the surgical procedures are given in the following section.

#### PECF group

The surgical procedures for PECF were similar to that previously published (13). Firstly, patients were placed prone with a mattress under their forehead, chest, and abdomen after receiving general anesthesia. The entry point was marked by C-arm, which was located on the superior border of the inferior lamina of the pathological segment near the medial of the facet joint on the herniated side. All instruments were from cervical foraminal system (Joimax, Germany). An 18-gauge puncture needle was inserted through the marked point until the inferior lamina was reached with the C-arm assistant. An incision around 8 mm was made above the V-point (the medial junction of the inferior and superior facet joint), and then a guidewire was placed and the needle withdrawn. Settings were as follows: 6.9 mm obturator, 8.0 mm oblique type working channel, and 4.1 mm endoscope. These were set by sequential dilation with continuous saline solution irrigation. Next, after identification of the V-point, the overlaying soft tissue was removed with endoscopic forceps. After the bone structure on the pathologic side was exposed, the lower margin of the superior lamina was drilled laterally toward the facet joint and caudally to the pedicle. Then, the intersection of the ascending facet with the inferior laminae was drilled with a radius around half of the facet length (6). The ligamentum flavum and foraminal ligament were removed so that the probe could easily insert into the foramen and dural sac and the outgoing nerve could be seen. The protruding nucleus pulposus, which is usually beneath the nerve root, was found and resected with tissue forceps. Finally, it was confirmed that the nerve root was completely decompressed before removing the working channel (14).

#### UBE group

**Figure 1:**
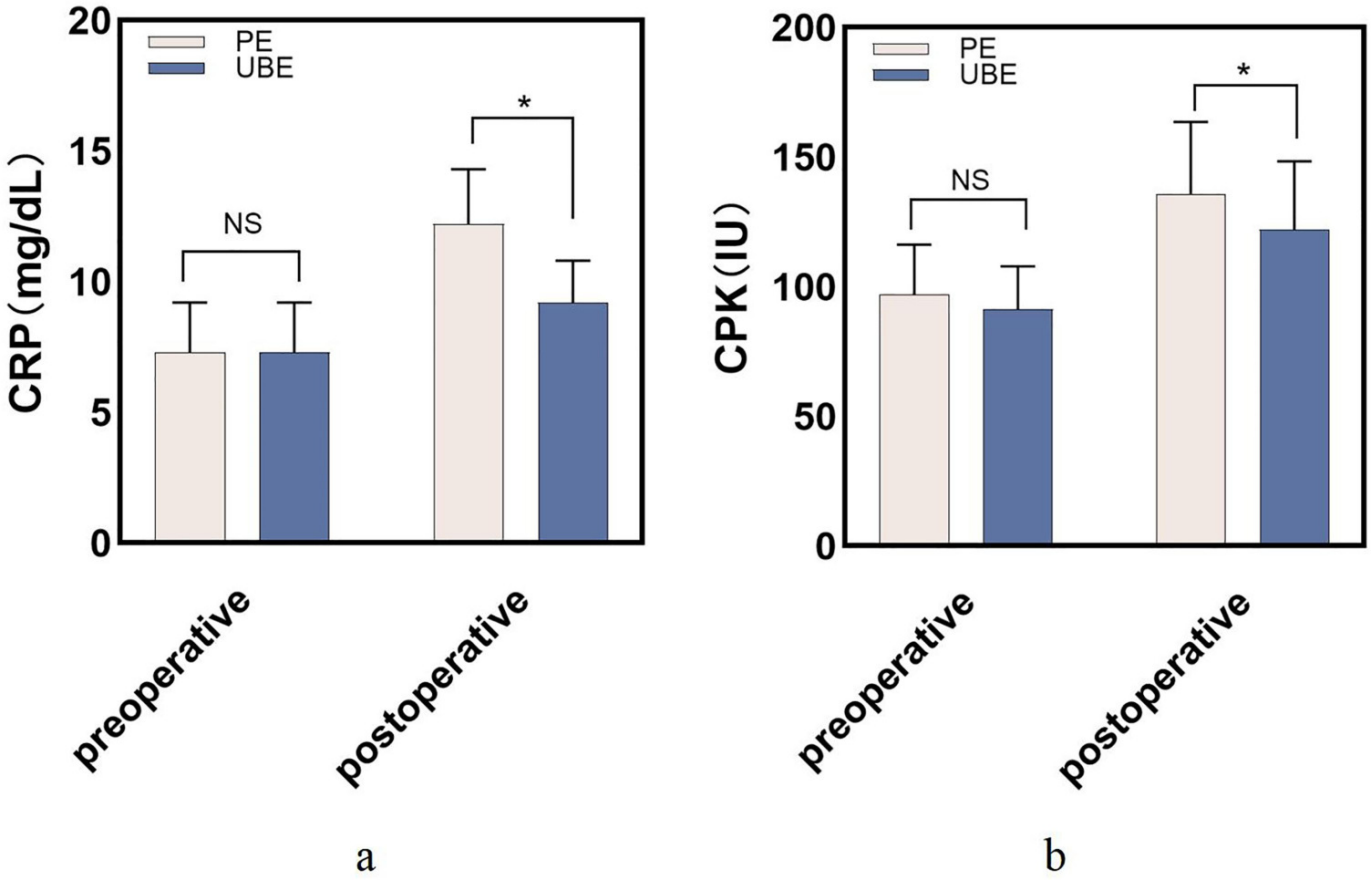
**(a)** Comparison of CRP levels between the two groups; **(b)** comparison of CPK levels between the two groups. *P < 0.05.

First, following the induction of general anesthesia, patients were placed in the prone position with a mattress under the forehead, chest, and abdomen. Under the guidance of the C-arm fluoroscope, the specific intervertebral disc space of the surgery segment was identified. The figure shown is a right side approach (Figure 1). At AP (anteroposterior) view, a vertical line was drawn along the outer edge of the upper vertebra's pedicle, and then a horizontal line was drawn at the junction where the lower vertebra's spinous process meets the lamina. 1 cm proximal and distal of the intersection points served as the observation and working channels. Under the C-arm, a puncture needle was inserted for the placement of skin dilators in sequence. After successfully establishing observation and working channels, a 30° endoscope was placed in the observation channel, and then the V point (intersection area of vertebral plates and the medial side of the facet joints) was found under the endoscope. This was confirmed before inserting into the surgery segment using the C-arm (15). At the lateral of the V point, the endoscopic drill was used to remove the portions of the superior and inferior vertebral plates as well as the medial aspect of the facet joint. Additional portions of the superior and inferior laminae were removed to ensure complete nerve root decompression if significant facet joint hyperplasia and severe nerve root compression were observed during surgery. The intervertebral foramen area was exposed, and the ligament flavum, corresponding nerve root, and the outer aspect of the dural sac were excised. A radiofrequency ablation probe was used for intervertebral disc ablation and hemostasis after the protruding nucleus pulposus were completely removed.

Postoperative rehabilitation was the same in both groups, and a neck collar was used for four weeks.

### Outcome evaluation

Baseline characteristics including age, BMI, gender, surgery level, and cost were collected from medical records, and operation-related parameters (Hospital stay and complications) and clinical outcomes such as Neck Disability Index (NDI) and Visual Analogue Scale (VAS) were also collected.

Radiological outcomes were measured by two experienced radiologists. The Cobb angle between the C2 and C7 vertebrae in the extension and flexion positions were measured.

As for clinical outcomes, neck pain was assessed using the Neck Disability Index (NDI) (16); the VAS was also used to analysis neck pain (VAS-n) and arm pain (VAS-a) (17), which was obtained preoperatively, immediately after operation, and at the 1-month follow-up visit. C-reaction protein (CRP) and serum creatine phosphokinase (CPK) were also analyzed, because these two indicators can accurately reflect the condition of soft tissue damage (18).

Complications were retrieved from the patients’ medical records and compared.Complications that adversely affected patient's recovery or required intervention, such as suppuration of the incision, leakage of the cerebrospinal fluid, intraspinal hematoma, and nerve root irritation symptoms, as well as redness, swelling of, or pain in the incision, were also recorded.

### Statistical analysis

Statistical Package for Social Sciences 24.0 (IBM Corp., Armonk, New York, USA) was used for the statistical analysis. All data were expressed as mean ± standard deviation. The Shapiro–Wilk test was used to determine the normality of continuous data. For comparisons between the two groups (UBE vs. PE), an independent samples *t*-test was used for normally distributed data (Bonferroni correction were used in multiple comparisons), and the Mann–Whitney *U*-test was used for non-normally distributed data. The categorical data were compared using the χ^2^ test. *P* < 0.05 was considered statistically significant.

## Result

### Baseline characteristics

We enrolled 123 CSR patients who met our criteria in this study and retrospectively reviewed their medical records (Table 1). There was no significant difference in age, gender, surgery level, blood loss, or pain side between the two groups (*P* > 0.05). However, hospital stay (2.1 ± 0.8 vs. 2.5 ± 0.5 days; *P* = 0.02) and operative time (61.1 ± 6.0 vs. 76.6 ± 8.3 min; *P* = 0.01) were significantly shorter in the UBE group than in the PE group. Moreover, patients in the UBE group achieved significantly shorter fluoroscopy than patients in PE group (6.9 ± 0.8 vs. 7.8 ± 0.8; *P* = 0.02. Additionally, the cost for the UBE group was significantly higher than for patients in the PE group (23,983.31 ± 1,184.88 CNY vs. 18,340.76 ± 2,098.17 CNY; *P* = 0.01).

**Table 1:** Comparison of characteristics between the two groups.

Group	UBE	PE	*p*-value
Number	60	63	
Gender			
Male	28	29	0.54
Female	32	34	
Age (year)	55.0 ± 9.0	57.3 ± 6.9	0.11
BMI (kg/m^2^)	23.0 ± 0.4	23.2 ± 0.4	0.06
Times of fluoroscopy	6.9 ± 0.8	7.8 ± 0.8	<0.05
Surgery level			
C3/4	7	9	0.92
C4/5	15	18	
C5/6	25	24	
C6/7	13	12	
Diabetes			
Yes	33	34	0.53
No	27	29	
Hospital stay (d)	2.1 ± 0.8	2.5 ± 0.5	<0.05
Cost (CNY)	23,983.31 ± 1,184.88	18,340.76 ± 2,098.17	<0.05
Blood loss (ml)	49.85 ± 5.8	51.25 ± 5.3	0.77
Operation time (min)	61.1 ± 6.0	76.6 ± 8.3	<0.05
Pain side			
Right	27	29	0.26
Left	33	34	

CNY, China Yuan; BMI, body mass index.

### Clinical and radiological outcomes

**Figure 2:**
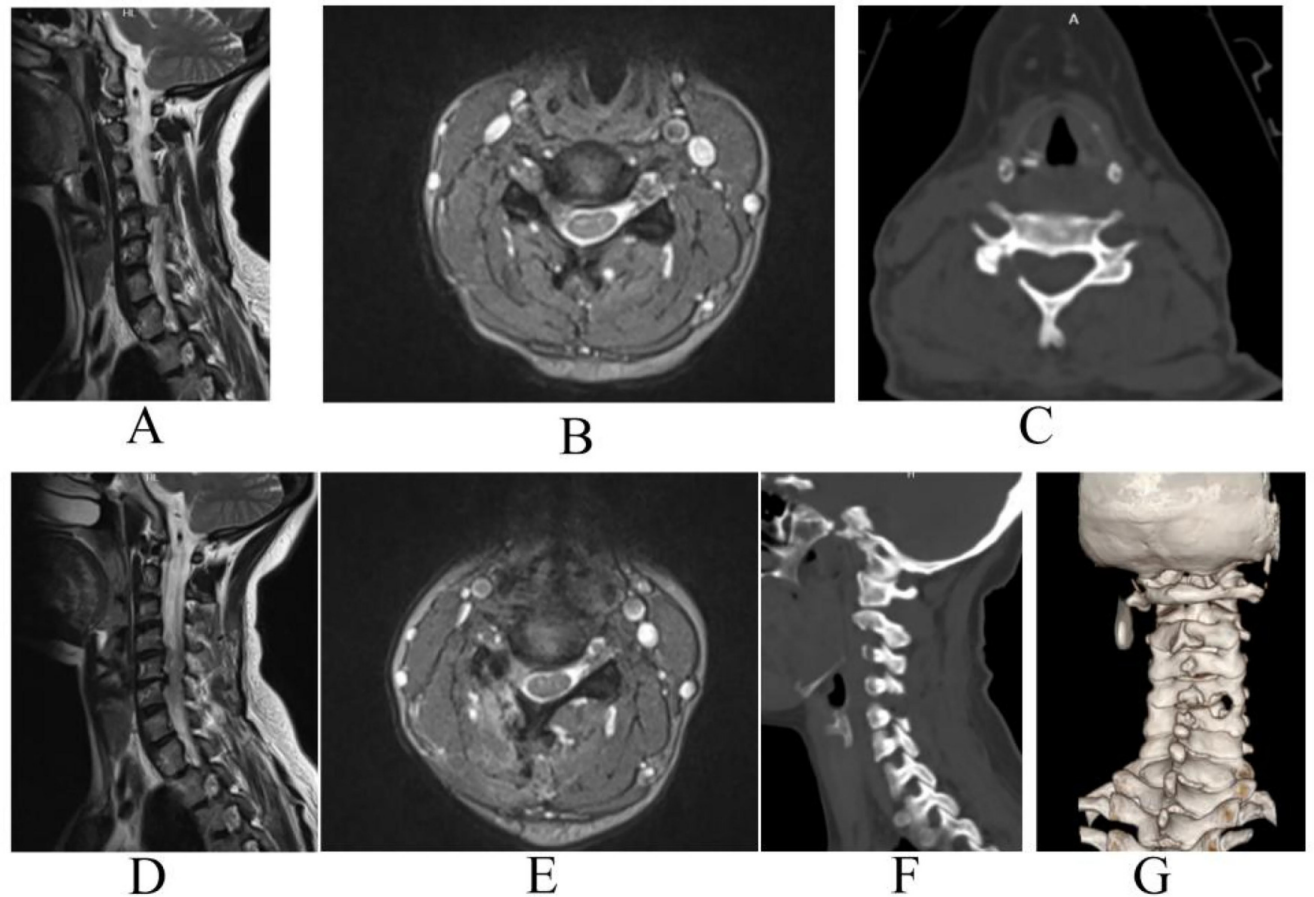
A 56-year-old female patient with C4/5 right cervical disc herniation who underwent UBE keyhole foraminotomy. **(A)** Sagittal view of MRI preoperatively; **(B)** transverse section view of MRI preoperatively; **(C)** transverse section view of CT postoperatively; **(D)** sagittal view of MRI postoperatively; **(E)** transverse section view of MRI postoperatively; **(F)** sagittal view of CT postoperatively; **(G)** 3D reconstruction of cervical spine postoperatively.

The clinical outcomes are shown in Table 2. There were no significant different on C2/7Cobb VAS and NDI between the two groups preoperatively (*P* > 0.05). During follow-up, the UBE group achieved significantly better VAS-N, VAS-A, and NDI scores than the PE group (*P* < 0.05). Additionally, the UBE group showed significantly lower CRP (9.2 ± 1.6 vs. 12.2 ± 2.1; *P* < 0.05) and CPK than the PE group 1 day after the operation (Figure 2).

**Table 2:** Comparison of clinical outcomes between the two groups.

Number	UBE	PE	*p*-value
	60	63	
Total C2/7Cobb			
Pre-operation	15.8 ± 1.7	15.7 ± 1.8	0.27
3-month follow-up	25.3 ± 1.6	23.9 ± 1.6	<0.05
1-year follow-up	24.9 ± 1.8	23.1 ± 1.9	<0.05
NDI			
Pre-operation	49.8 ± 3.0	50.0 ± 6.3	0.27
1-month follow-up	16.5 ± 2.1	19.2 ± 3.7	<0.05
1-year follow-up	10.0 ± 1.4	12.0 ± 2.2	<0.05
VAS-N			
Pre-operation	5.6 ± 1.1	5.7 ± 1.0	0.86
Immediately after operation	3.3 ± 0.7	3.7 ± 1.1	<0.05
1-month follow-up	1.5 ± 1.2	2.2 ± 0.8	<0.05
VAS-A			
Pre-operation	5.1 ± 0.8	5.0 ± 0.8	0.9
Immediately after operation	3.3 ± 0.8	3.7 ± 0.5	<0.05
1-month follow-up	0.9 ± 0.8	1.5 ± 1.1	<0.05
CRP			
Pre-operation	7.2 ± 1.7	7.3 ± 1.9	0.85
1 day after operation	12.2 ± 2.1	9.2 ± 1.6	<0.05
CPK			
Pre-operation	97.0 ± 19.3	91.3 ± 16.6	0.08
1 day after operation	122.1 ± 26.3	135.9 ± 27.7	<0.05

NDI, neck disability index; VAS-N, visual analogue scale of neck; VAS-A, visual analogue scale of arm; CRP, C-reaction protein; CPK, serum creatine phosphokinase.

As for complications and the MacNab criteria (Table 3), in the UBE group, there were two patients were incision exudation, one with nerve root irritation, and one with wound and pulmonary infection. In the PE group, five cases of incision exudation occurred, three patients had nerve root irritation, four experienced mild spinal epidural tear, and three patients suffered from wound infection, which was successfully managed by conservative treatment. According to the MacNab criteria, the overall excellent and good rate was 96.67% (29/30) in the UBE group and 92.06% (58/63) in the PE group.

**Table 3:** The comparison of complications and Macnab criteria between thetwo groups.

Group	UBE	PE	*p*-value
Complication			
Incision exudation	2	5	<0.05
Nerve root irritation	1	3	
Mild spinal epidural tear	0	4	
Venous thromboembolism	0	2	
Wound and pulmonary infection	1	3	
MacNab			
Excellent	46	40	<0.05
Good	12	18	
Middle	1	4	
Poor	1	1	

## Discussion

Daily work activities often place increased stress on the cervical spine, leading to a higher incidence of cervical spondylotic radiculopathy (CSR).CSR is characterized as the degeneration of the intervertebral disc, facet joint, posterior longitudinal ligament, and ligamentum flavum, which leads to compression or irritation of the nerve root and secondary inflammatory (19). Surgical intervention is necessary for around 10%–25% patients whose symptoms do not improve after conservative treatment (20). Surgical treatment may be needed for complete decompression and to avoid degenerative progression, which conservative treatment cannot achieve.

Percutaneous endoscopic cervical keyhole foraminotomy (PE) is a widely used minimally invasive technique for patients with CSR (21). However, recurrence and occasional pain have been reported with this technique. Ruetten et al. carried out a two-year follow-up retrospective study about endoscopic posterior foraminotomy in the treatment of cervical lateral disc herniation, and found that 87.4% of patients no longer had arm pain and 9.2% had only occasional pain. Although no serious surgical complications occurred, 3.4% suffered from recurrence during the follow-up visit period (22). As a result, unilateral biportal endoscopy technique was proposed due to its more flexible instruments involved in operation, better decompression, and less intraoperative fluoroscopy times than PE (8). Park et al. reported on the UBE technique in the treatment of cervical spine using 14 patients with CSR who underwent posterior percutaneous endoscopic cervical discectomy with a unilateral biportal endoscopic approach. These patients achieved significant improvement in VAS and NDI postoperatively, demonstrating this could be a good alternative approach for cervical disc herniation and foraminal stenosis (23). Moreover, Song et al. reported that the UBE technique can achieve sufficient decompression of the foraminal area by removing nearly 50% of the facet joint bone without affecting cervical stability. And UBE may also minimize iatrogenic damage to the posterior cervical musculo-ligamentous structure and maximize the preservation of the facet joint to maintain cervical stability (10).

In this study, both the PE and UBE groups underwent a keyhole technique involving limited paraspinal muscle dissection.We compared the clinical outcomes of 60 (UBE group) and 63 patients (PE group), and there were no significant differences in age, gender, surgery level, or pain side between the two groups (*P* > 0.05). During follow-up, significantly shorter hospital stay (2.1 ± 0.8 day vs. 2.5 ± 0.5 day; *P* < 0.05), higher cost (23,983.31 ± 1,184.88 CNY vs. 18,340.76 ± 2,098.17 CNY; *P* < 0.05), shorter operation time (61.1 ± 6.0 min vs. 76.6 ± 8.3 min; *P* < 0.05), and shorter fluoroscopy times (6.9 ± 0.8 vs. 7.8 ± 0.8; *P* < 0.05) were reported in UBE group. Similar outcomes were reported by Tang et al. (12). Wang et al. also reported that the average total hospitalization cost of the UBE group was significantly higher than for patients in the PE group (24.09 ± 2.44 thousand RMB vs. 18.62 ± 2.87 thousand RMB; *P* < 0.05) and that they experienced significantly lower fluoroscopy times as well (24). Further, Kang et al. reported on 65 patients with single-level unilateral cervical foraminal disc disease who underwent UBE (*n* = 33) or PE (*n* = 32) and found that the operation time was significantly shorter in the UBE group than the PE group (25).

Since both PE and UBE are minimally invasive techniques, CRP and CPK levels were also analyzed to **assess** soft tissue damage during the surgery. In our study, we found that the UBE group showed significantly lower CRP and CPK than the PE group 1 day after the operation. Similarly, Park et al. reported on 100 patients who underwent UBE (*n* = 50) or microdiscectomy (*n* = 50) for lumber disc herniation and found that patients in the UBE group achieved significantly lower serum creatine phosphokinase (CPK) levels, which indicates better muscle preservation (26). As for comparison of clinical outcomes, there were no significant differences in Cobb C2/C7, NDI, VAS, or CRP between the two group preoperatively , however significantly better clinical outcomes were observed during the follow-up visit period (*P* < 0.05). One possible reason could be that, during UBE, surgeons can precisely decompress of the affected nerve, which involves procedures such as discectomy and foraminotomy (27). 2. The integrity of the dorsal neck musculature and ligamentous complexes were better preserved, which is essential for preventing post-operative cervical instability and axial cervical discomfort (12). 3. The PE technique is a uniportal long-axis technique that poses several challenges due to its unique design, such as combining both work and view functions within the same narrow portal, which may be disorientating and creature difficulty in maneuvering. Regarding complications and MacNab, there were significantly more patients who suffered from complications in the PE group than in the UBE group. Excellent and good MacNab scores in the UBE group were 96.67% (29/30), while they were 92.06% (58/63) in the PE group.

## Conclusion

Both PE and UBE are safe and effective methods for treating cervical spondylotic radiculopathy. UBE can provide shorter operation times, low CRP levels, and better muscle preservation than PE. However, further prospective studies need to be taken to ascertain whether UBE can better preserve muscle tissue.

## Limitations

This study has several limitations. First, there is a potential risk of selection bias due to it being a non-randomized and single-center, small-sample, retrospective study. Second, more indicators (such as MRI outcome) about muscle tissue damage need be take into account to evaluate the effect of the two techniques on soft tissue. Further studies are needed to confirm the effectiveness of UBE and PE in the treatment of cervical spondylotic radiculopathy.

## Data Availability

The raw data supporting the conclusions of this article will be made available by the authors without undue reservation.
